# *PLEKHG1*: New Potential Candidate Gene for Periventricular White Matter Abnormalities

**DOI:** 10.3390/genes15081096

**Published:** 2024-08-20

**Authors:** Francesco Calì, Mirella Vinci, Simone Treccarichi, Carla Papa, Angelo Gloria, Antonino Musumeci, Concetta Federico, Girolamo Aurelio Vitello, Antonio Gennaro Nicotera, Gabriella Di Rosa, Luigi Vetri, Salvatore Saccone, Maurizio Elia

**Affiliations:** 1 Oasi Research Institute—IRCCS, 94018 Troina, Italy; cali@oasi.en.it (F.C.); mvinci@oasi.en.it (M.V.); streccarichi@oasi.en.it (S.T.); cpapa@oasi.en.it (C.P.); agloria@oasi.en.it (A.G.); amusumeci@oasi.en.it (A.M.); avitello@oasi.en.it (G.A.V.); lvetri@oasi.en.it (L.V.); melia@oasi.en.it (M.E.); 2 Department Biological, Geological and Environmental Sciences, University of Catania, Via Androne 81, 95124 Catania, Italy; concetta.federico@unict.it; 3 Unit of Child Neurology and Psychiatry, Department of Human Pathology of the Adult and Developmental Age, “Gaetano Barresi” University of Messina, 98124 Messina, Italy; antonionicotera@ymail.com (A.G.N.); gdirosa@unime.it (G.D.R.)

**Keywords:** periventricular leukomalacia, white matter hyperintensities, cell division control protein 42 (CDC42), GTPase pathway, next-generation sequencing, whole exome sequencing

## Abstract

Hypoxic-ischemic brain damage presents a significant neurological challenge, often manifesting during the perinatal period. Specifically, periventricular leukomalacia (PVL) is emerging as a notable contributor to cerebral palsy and intellectual disabilities. It compromises cerebral microcirculation, resulting in insufficient oxygen or blood flow to the periventricular region of the brain. As widely documented, these pathological conditions can be caused by several factors encompassing preterm birth (4–5% of the total cases), as well single cotwin abortion and genetic variants such as those associated with GTPase pathways. Whole exome sequencing (WES) analysis identified a de novo causative variant within the pleckstrin homology domain-containing family G member 1 (*PLEKHG1*) gene in a patient presenting with PVL. The *PLEKHG1* gene is ubiquitously expressed, showing high expression patterns in brain tissues. *PLEKHG1* is part of a family of Rho guanine nucleotide exchange factors, and the protein is essential for cell division control protein 42 (CDC42) activation in the GTPase pathway. CDC42 is a key small GTPase of the Rho-subfamily, regulating various cellular functions such as cell morphology, migration, endocytosis, and cell cycle progression. The molecular mechanism involving PLEKHG1 and CDC42 has an intriguing role in the reorientation of cells in the vascular endothelium, thus suggesting that disruption responses to mechanical stress in endothelial cells may be involved in the formation of white matter lesions. Significantly, CDC42 association with white matter abnormalities is underscored by its MIM phenotype number. In contrast, although PLEKHG1 has been recently associated with patients showing white matter hyperintensities, it currently lacks a MIM phenotype number. Additionally, in silico analyses classified the identified variant as pathogenic. Although the patient was born prematurely and subsequently to dichorionic gestation, during which its cotwin died, we suggest that the variant described can strongly contribute to PVL. The aim of the current study is to establish a plausible association between the *PLEKHG1* gene and PVL.

## 1. Introduction

Brain white matter, a complex network of myelinated nerve fibers, is responsible for transmitting signals across diverse brain regions. It enables the establishment of the brain communication network, facilitating rapid information processing and coordination of cognitive functions [1,2]. Among the broad spectrum of phenotypes associated with brain white matter dysregulations, periventricular leukomalacia (PVL) is a crucial disease. Particularly, it is characterized by injury to the white matter surrounding the brain ventricles, often occurring in premature infants. The vulnerability of developing white matter to hypoxic-ischemic insults during the perinatal period increases the risk of PVL [2,3,4,5]. Additionally, it was frequently associated with cognitive and motor impairments due to disruptions in the transmission of neural signals within the affected white matter tracts [6,7]. The severity of symptoms and their impact on a child’s functioning are influenced by the extent and specific location of the lesions, which can significantly hinder normal development and lead to long-term disabilities [8]. The most common symptom is cerebral palsy, a condition that affects coordination and movement, but children with periventricular leukomalacia may also have intellectual disability, eye movements disorders, hearing loss and vision problems, learning disabilities, seizures, and urinary incontinence. Significantly higher white matter lesion density in the left superior longitudinal fasciculus and the right frontal projections of the corpus callosum of patients have been linked to psychiatric symptoms, as these tracts are crucial for cognitive and emotional functions [9]. In fact, advanced imaging techniques allow for the detection and monitoring of white matter lesions, aiding in the diagnosis and management of PVL-related complications.

As recently discussed, further exploration of the genetic underpinnings of white matter hyperintensities is crucial for a comprehensive understanding of associated symptoms and diseases. These lesions, whose pathophysiology remains elusive, likely stem from processes such as demyelination, gliosis, and axonal loss, possibly triggered by chronic cerebral hypoperfusion or compromised blood–brain barrier integrity [10]. White matter abnormalities have been linked to the dysregulation of GTPase activity, which potentially disrupts cellular processes crucial for white matter integrity [11,12,13]. Understanding the intricate relationship between GTPase signaling pathways and white matter abnormalities could provide insights into the pathogenesis of neurological disorders. The GTPase cycle involves the conversion of GTP to GDP and vice versa by GTPases, such as the Rho family proteins, regulating various cellular processes. GTPases alternate between an active GTP-bound state and an inactive GDP-bound state, modulating downstream signaling pathways critical for cell function and homeostasis.

Within this context, Pleckstrin homology domain-containing family G member 1 (PLEKHG1) is a Rho guanine nucleotide exchange factor that is involved in reorientation of cells in the vascular endothelium [14]. As demonstrated, it is engaged in the cyclic stretch-induced reorientation of human umbilical vein endothelial cells (HUVECs) and their stress fibers in a direction perpendicular to the stretch axis [14]. The Rho guanine nucleotide exchange factor PLEKHG1 is activated by interaction with and phosphorylation by Src family kinase member FYN [15]. It is well-established that Rho family small GTPases (Rho) play a crucial role in regulating diverse cell motility processes by intricately controlling the actin cytoskeleton in a spatiotemporal manner [15]. Certain Rho-specific guanine nucleotide exchange factors (RhoGEFs) undergo regulation through tyrosine phosphorylation by Src family tyrosine kinases (SFKs). Within this context, PLEKHG1 interacts with cell division control protein 42 (CDC42) in the GTPase cycle, facilitating its activation through the exchange of GDP for GTP. This interaction is mediated by the DH domain of PLEKHG1, which acts as a guanine nucleotide exchange factor. Upon activation, CDC42 regulates diverse cellular processes such as cell morphology, migration, and signaling cascades, contributing to overall cellular homeostasis and function [15,16]. Within this context, CDC42 is also activated by pleckstrin homology domain-containing family G member 2 (PLEKHG2), RhoGEF and PH domain-containing protein 1 (FGD1), and phosphatidylinositol 3,4,5-trisphosphate-dependent Rac exchanger 1 protein (pRex1), with the DH domain pivotal in this process. Particularly, the DHPH tandem domains, also found in PLEKHG1, exhibit diverse regulatory roles, including localization, autoinhibition, and interactions with proteins or lipids, as demonstrated in various studies [15,16]. As recently documented, genetic variation within the *PLEKHG1* gene was associated with white matter hyperintensities [17]. Furthermore, a specific mutation of the *PLEKHG1* gene was associated with preeclampsia [18].

Herein, we present a clinical case study of a patient displaying PVL as an outcome of a perinatal hypoxic-ischemic event. Our primary objective is to establish a potential causal link between the genetic variant identified within *PLEKHG1* and PVL, which could significantly contribute to our understanding of the genetic underpinnings of white matter dysregulations.

## 2. Materials and Methods

### 2.1. Library Preparation and Next-Generation Sequencing (NGS)

Genomic DNA was retrieved from the patient’s and parents’ peripheral blood leukocytes. DNA extraction was performed in alignment with a specific protocol, previously described [19,20,21]. Library preparation (TRIOS) and exome enrichment were carried out using the Agilent SureSelect V7 kit (Santa Clara, CA, USA) following the manufacturer’s instructions. A sequencing run was performed on an Illumina HiSeq 3000 instrument (San Diego, CA, USA). This approach enabled the achievement of 97% of regions covered at least 20×. The identified variants were filtered based on (i) recessive/de novo/X-linked pattern of inheritance, (ii) allele frequencies (mean average frequency, MAF) <1% using as reference the following genomic datasets: 1000 Genomes, ESP6500, ExAC, gnomAD. The confirmation of the de novo event was achieved through conventional Sanger sequencing using the BigDyeTM Terminator v1.1 Cycle Sequencing Kit (Life Technologies, CA, USA) with the SeqStudio Genetic Analyzer instrument (Thermo Fisher Scientific, Waltham, MA, USA). Primers were for. 5′-GAGAGTGGACTCAAACGGGC-3′, rev. 5′- CATCCCCTACACCTGGCTTG-3′. According to a previous protocol [22], the patient’s and parents’ DNA fingerprint analysis was performed to confirm maternity and paternity.

### 2.2. Data Analysis

The variant was searched on the Human Gene Mutation Database (HGMD Professional 2023). Several filters were employed using VarAft (2.17-2) [23]. The identified variant was classified in alignment with the “American College of Medical Genetics” (ACMG) guidelines [24] throughout VarSome (Release 11.16), according to a previous study [25]. Table S1 shows the ACMG criteria employed for variant classification as likely pathogenic. Multiple algorithms were employed for the variant classification, including PolyPhen 2 (http://genetics.bwh.harvard.edu/) (accessed on 3 June 2024) and Mutation Taster (https://www.mutationtaster.org/) (accessed on 3 June 2024). The Human Protein Atlas (HPA) (https://www.proteinatlas.org/) (accessed on 3 June 2024) was consulted to investigate the *PLEKHG1* expression patterns. Uniprot database (https://www.uniprot.org/) (accessed on 3 June 2024) was used for retrieving the PLEKHG1 protein details related to the wild-type protein sequence and functional domain organization. The graphical representation of the amino acid chain flanking the identified variants was obtained from the UCSC Genome Browser database (https://genome.ucsc.edu) (accessed on 3 June 2024). Gene ontology (GO) terms related to the protein and functional domain annotations were obtained from the QuickGO database (https://www.ebi.ac.uk/QuickGO/) (accessed on 3 June 2024). Protein structure predictions were generated employing UCSF ChimeraX software version 1.7 (software developed by the Resource for Biocomputing, Visualization, and Informatics at the University of California, San Francisco, with support from National Institutes of Health R01-GM129325 and the Office of Cyber Infrastructure and Computational Biology, National Institute of Allergy and Infectious Diseases) (https://www.cgl.ucsf.edu/chimerax/) (accessed on 3 June 2024). In particular, this software utilizes the AlphaFold algorithm generating five protein models, and in accordance with its output, the “best model” was selected for our investigation. The selection of the best model was carried out by the AlphaFold algorithm based on the predicted alignment error illustrated in Figure S1. Additionally, the MuPRO tool was used for estimating the impact of the mutations on protein stability (http://mupro.proteomics.ics.uci.edu) (accessed on 3 June 2024) by a score predicting the value and sign of energy change (delta delta G), spanning from −1 to 1 [26]. The interaction between PLEKHG1 and CDC42 was analyzed using Pipeline for the Extraction of Predicted Protein-protein Interactions (PEPPI) algorithm (https://seq2fun.dcmb.med.umich.edu/PEPPI/) (accessed on 14 August 2024). As documented, this web server provides a logarithmic variation in the likelihood ratio (logLR) score [27]. KEGG (https://www.kegg.jp/) (accessed on 3 June 2024) and STRING (version 12.0) databases (https://string-db.org/) (accessed on 3 June 2024) were used to investigate the molecular pathways involving the target PLEKHG1 protein.

The Human Phenotype Ontology (HPO) database (https://hpo.jax.org/) (accessed on 3 June 2024) was consulted to investigate the phenotypes typically associated with cystic fibrosis. This investigation aimed to demonstrate that the patient’s symptoms are inconsistent with those commonly observed in cystic fibrosis.

## 3. Results

### 3.1. Clinical Report

The patient is a female who was born prematurely at 34 weeks and 6 days from a dichorionic diamniotic pregnancy, with her twin being stillborn at 28 weeks gestation. An emergency Cesarean section was performed due to premature rupture of membranes and the expulsion of the twin sac. The patient’s birth weight was 2090 g, and her Apgar score was within the normal range. At one year of age, the patient was diagnosed with psychomotor delay and suspected hypertonia in the lower limbs. In light of these findings, the child was referred to the Oasi Research Institute—IRCCS (Troina) for further evaluation and management.

Neurological examination at the age of 13 months showed axial hypotonia with signs of hypertonia in the lower limbs, presenting equinism of the feet and evident resistance to dorsiflexion of the tibiotarsal joint; moreover, proximal and distal crossing tendencies were observed. There was no increase in muscle tone in the upper limbs. Furthermore, upon verticalization, the patient demonstrated a hypovalid suspension reaction. The osteotendinous reflexes exhibited brisk and symmetrical responses. No foot clonus was elicited, though an extension of the plantar reflex persisted. Additionally, the patient presented with sialorrhea associated with deficits in orobuccal skills. The language was restricted to babbling and vocalizations.

Electroencephalogram, electrocardiogram, eye examination, abdominal ultrasound, as well as metabolic blood and urine screening results were within the normal range.

Brain MRI revealed diffuse and marked T2 hyperintensity in the periventricular white matter and semioval centers, with extensive involvement of the tapetum. Irregularities and festooning of the walls of the lateral ventricles were noted, especially in the regions of the medial cells and trigones, with modest ventricular cavity constriction. The corpus callosum was significantly thinned, especially in the posterior third of the body and splenium, suggesting periventricular leukomalacia.

Consequently, a diagnosis of “Spastic diplegia and psychomotor developmental delay” was made, and a psychomotor rehabilitation treatment was recommended.

### 3.2. Next Generation Sequencing (NGS)

NGS did not identify genetic variants in known genes related to the patient’s phenotype. However, the patient was compound heterozygous for two causative, previously described mutations (c.202A>G:p.K68E and c.1520_1522del:p.507_508del) within cystic fibrosis transmembrane conductance regulator (*CFTR*) gene, associated with cystic fibrosis (NM_000492) [28,29]. The two CFTR variants, already documented in the literature, had been inherited from both the mother and the father, resulting in a compound heterozygosity condition in the examined patient.

**Figure 1:**
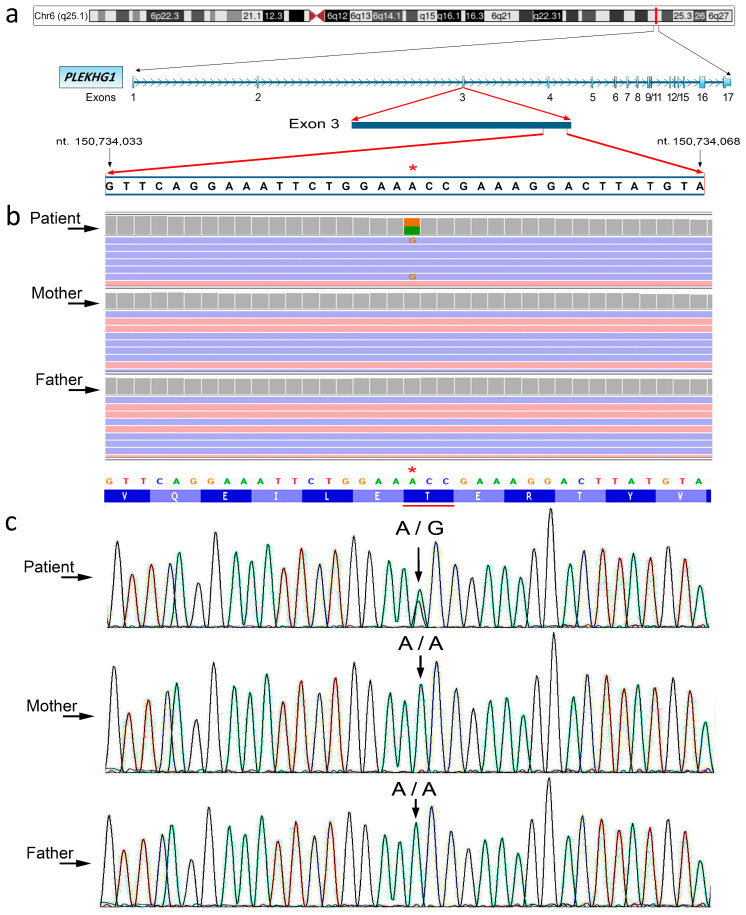
Detection of the *PLEKHG1* gene variant. (**a**) From upper to bottom: ideogram of the human chromosome 6 with the position of the *PLEKHG1* gene in the chromosomal band 6q25.1. Exon/intron organization of the *PLEKHG1* gene, and transcriptional direction. Highlight of the nucleotide sequence from the exon 3 shown in (**b**,**c**). The position of the first and last nucleotide (nt) here considered are indicated. The red asterisk indicates the observed nucleotide variant. Data from UCSC genome browser, human GRCh/hg38 (https://genome.ucsc.edu, accessed on 3 June 2024). (**b**) WES results for the examined patient and the healthy parents. Data are presented using the Integrative Genomics Viewer (IGV) visualization tool. (**c**) Sanger sequencing to highlight the variant c.370A>G identified by WES. The amino acid sequence is shown below the nucleotide sequence. The red asterisk indicates the nucleotide variant. The underlined amino acid is the affected position. The genotype for each subject is shown.

Nevertheless, WES Trio analysis unveiled a heterozygous de novo mutation (c.370A>G) in exon 2 of the *PLEKHG1* gene (NM_001029884.3). The variant was a missense mutation leading to the amino acid change of Thr with Ala at position 124 (p.Thr124Ala) of the protein (NP_001025055.1) (Figure 1). The variation identified in *PLEKHG1* was localized within the Dbl homology (DH) domain (from aa 113 to aa 293). In silico analysis, carried out employing diverse algorithms, described the variant as likely pathogenic (Table 1).

As reported in Table 1 and illustrated in Figure S2, several tools classified the variant as damaging. According to the GnomAD database, this variant shows a very low allele frequency of ƒ = 0.000000684 among the worldwide population (1,461,698 individuals). The variant was confirmed through conventional Sanger sequencing (Figure 1).

**Figure 2:**
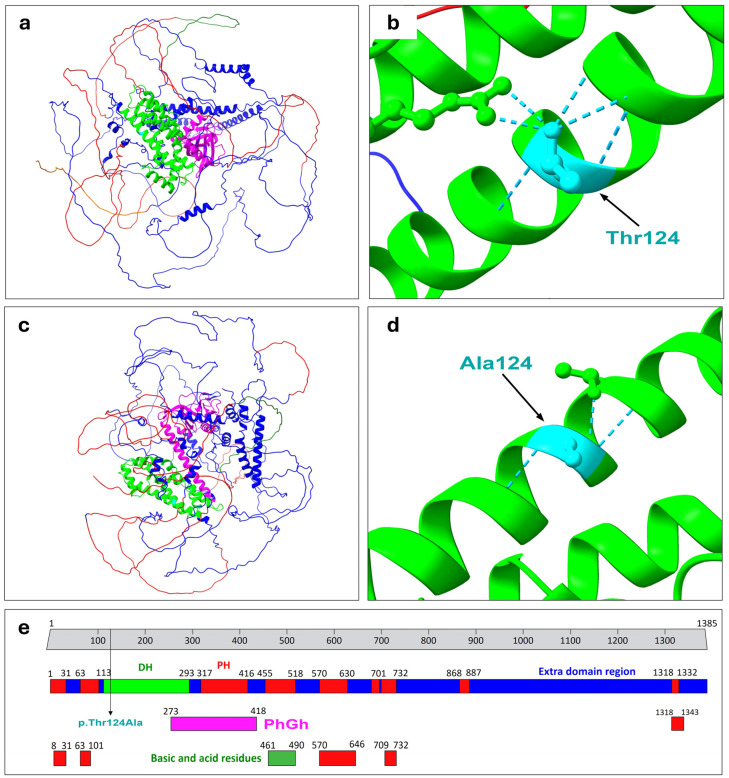
Structure prediction analysis related to the wild-type and mutated PLEKHG1 protein. (**a**) Wild-type structure prediction of PLEKHG1 protein. (**b**) Focus on the wild-type Threonine (Thr) residue at position 124. The six hydrogen bonds (2 with Arg251, 2 with Glu120, 1 with Ile121, and 1 with Tyr128) are depicted in light blue. (**c**) Mutated PLEKHG1 protein structure prediction, which differs from the wild type as result of the diverse number of rearrangements of the hydrogen bonds as a consequence of the genetic variation. (**d**) Focus on the mutated Alanine residue at position 124. The three hydrogen bonds (Glu120, Thr127 and Tyr128) are depicted in light blue. Figures (**a**–**d**) were generated employing UCSF ChimeraX. (**e**) Graphical representation of the organization of PLEKHG1 functional domains and regions. The Figure was modified from Uniprot database. PhGh: Pleckstrin homology domain-containing family G members 1, 2, and 3 pleckstrin homology (PH) domain.

The protein structure prediction related to both the wild-type and mutated PLEKHG1, carried out using UCSF ChimeraX, enabled the identification of notable differences between the mutated and the wild-type protein (Figure 2).

UCSF ChimeraX protein structure prediction accounted for a total of 651 hydrogen bonds for the wild-type (WT) PLEKHG1. Within this context, the wild-type Threonine residue exhibited a total number of six hydrogen bonds: two with Arg251, two with Glu120, one with Ile121, and one with Tyr128. Differently, the mutated PLEKHG1 displayed a total of 479 hydrogen bonds (loss of 172 bonds compared to the WT), while the mutated Alanine residue at position 124 accounted for three hydrogen bonds, specifically with Glu120, Thr127 and Tyr128.

MuPRO analysis unveiled a decreased protein stability as a result of the mutation, assigning a delta delta G score of −0.61471774 (spanning from −1 to 1). MutPred2 classified the variant as probably pathogenic, with a score of 0.606. Moreover, it suggested potential protein structure alterations, including changes to the coiled-coil domain, the acquisition of an allosteric site at Arginine 126, or potential modifications to the metal-binding site. STRING analysis, based on experimental evidence, indicated a robust protein–protein interaction with CDC42 protein and RAC1. Within this context, the PEPPI algorithm predicted a reduced interaction between the mutated PLEKHG1 and CDC42, with a score of 1.072, compared to a score of 1.083 for the wild-type PLEKHG1.

## 4. Discussion

We report the case of a child, born from healthy parents, showing PVL and perinatal hypoxic ischemia. She survived a dichorionic diamniotic gestation, during which her cotwin experienced spontaneous abortion during the fetal stage. As was extensively reported, a high incidence of cerebral palsy has been observed in twins born subsequent to a spontaneous abortion. Nevertheless, the mechanism underlying cerebral palsy in twins who survived dichorionic gestations remains unknown [30,31]. The rate of twins affected by intellectual disability and cerebral palsy is six times higher in monochorionic gestations compared to dichorionic ones [32,33,34]. Additionally, the child was born prematurely, a factor that may cause PVL, potentially affecting neurodevelopmental processes [35,36]. Although these factors can contribute to PVL, we intended to highlight the role of the genetic variant observed in this study as concomitant cause, even considering the dichorionic gestation. Furthermore, it has been demonstrated that only 4–5% of infants born prematurely develop PVL [37]. Among these, 20–30% are born between the 24th and 26th week of gestation and have a birth weight of less than 1 kg. In contrast, the patient here reported was born at 34 weeks of gestation with a birth weight of 2.090 kg.

Brain white matter comprises myelinated nerve fibers that are essential for transmitting signals between brain regions, facilitating rapid information processing and cognitive function coordination. Dysfunction in white matter significantly impairs neural communication, resulting in cognitive deficits and neurological disorders [38,39]. The vulnerability of developing white matter to hypoxic-ischemic insults during the perinatal period increases the risk of PVL [2,3,4,5]. Additionally, it has been frequently associated with cognitive and motor impairments due to disruptions in the transmission of neural signals within the affected white matter tracts [6,7].

In the clinical case examined in this study, WES Trio analysis unveiled a de novo genetic variant within *PLEKHG1*, a gene previously correlated with white matter hyperintensities, through an association study [17]. This gene is intricately engaged in the guanine nucleotide exchange factor, which operates in reorientating vascular endothelium cells [14,16]. Additionally, it is predicted to exhibit guanyl-nucleotide exchange factor activity and plays a role in regulating small GTPase-mediated signal transduction. The protein is primarily located in the nucleoplasm and governs diverse cellular processes, including cytoskeletal organization and signal transduction pathways crucial for cell motility and morphology [15,16,40].

NGS analysis revealed two heterozygous mutations within the *CFTR* gene, known for its causative effect in cystic fibrosis. This condition affects multiple organs, including the exocrine pancreas, contributing to cystic fibrosis-related diabetes [41,42]. Cystic fibrosis is associated with a wide range of symptoms, as the Human Phenotype Ontology (HPO) indicates. However, the periventricular leukomalacia observed in the patient does not align with these symptoms. However, it cannot be ruled out that cystic fibrosis may manifest in the future, as the genomic test indicates. Multiple algorithms classified the variant identified in the examined patient as likely pathogenic (Table 1). Specifically, it was localized within the Dbl homology (DH) domain (from aa 113 to aa 293). This functional domain is high conserved, as indicated by the sequence coverage analysis and the plDDT score illustrated in Figure S3 and Figure S4, respectively. As documented, this domain plays an essential role serving as a highly efficient catalytic machine, exponentially accelerating the nucleotide exchange of Rho proteins. It is frequently accompanied by a pleckstrin homology (PH) domain, underscoring its indispensable and conserved role [43,44]. Within the DBL family proteins, the DH domain exclusively executes the catalytic guanine nucleotide exchange activity, not only being sufficient for this function but also crucial for substrate specificity [45,46,47]. In particular, it was demonstrated that the DH domain within PLEKHG1 is intricately linked with CDC42, being important for its activation [15].

The protein structure prediction analysis carried out with UCSF ChimeraX indicated a notable variation in the hydrogen bonds within the protein structure. Specifically, the mutated PLEKHG1 accounted for a loss of 172 hydrogen bonds compared to the wild-type protein. Additionally, the wild-type Threonine residue at position 124 displayed different hydrogen bond patterns compared to the mutated Alanine 124 identified in the patient. Furthermore, the MuPRO tool indicated a decreased stability of the mutated protein. Particularly, MutPred2 classified the variant as probably pathogenic (score: 0.606) and predicted potential protein alterations encompassing notable changes in the coiled-coil domain, the gain of an allosteric site in correspondence with the Arginine residue at position 126, or modifications to the metal-binding site. Previous studies indicated that alteration of the coiled-coil domain can significantly impact protein structure, altering its stability and structural specificity and exerting a broad array of biological implications [48,49]. Allosteric sites are regions on a protein surface distinct from the active site where regulatory molecules can bind, influencing the protein’s activity and its conformational equilibrium [50,51]. As documented, PLEKHG1 shows ubiquitarian expression patterns, encompassing diverse brain tissues [52,53].

As concerns the molecular pathways encompassing the PLEKHG1 protein, a KEGG orthology (KO) term was assigned (K23859). According to gene ontology (GO) annotations from QuickGO, PLEKHG1 is associated with molecular functions such as guanyl-nucleotide exchange factor activity (GO:0005085) and small GTPase binding (GO:0031267). It plays a role in regulating small GTPase-mediated signal transduction (GO:0051056). Regarding cellular localization, PLEKHG1 is found in the cytosol (GO:0005829) and nucleoplasm (GO:0005654) according to the Reactome and Human Protein Atlas databases, respectively. Additionally, Reactome indicates PLEKHG1 involvement in the GTPase cycle associated with CDC42 (R-HSA-9013148) and RAC1 (R-HSA-9013149) proteins. In that framework, STRING analysis revealed a strong association with CDC42, a gene recently associated with diverse neurodevelopmental phenotypes including white matter anomalies [54]. It is noteworthy that CDC42 has been assigned to a MIM phenotype number (616737) associated with Takenouchi–Kosaki syndrome, characterized by diverse symptoms like delayed psychomotor development, learning disability, poor or absent speech, and white matter abnormalities, thin corpus callosum, and periventricular white matter abnormalities mirroring those observed in the patient under examination. PLEKHG1 is intricately involved in the GTPase cycle of CDC42, playing a crucial role in its activation [15]. Specifically, CDC42 is strongly activated by four genes (PLEKHG2, PLEKHG1, pRex1, and FGD1), with the DH domain playing a pivotal role in its activation. Indeed, studies have shown that the DHPH tandem domains (also present within PLEKHG1) can have various regulatory functions, including localization, autoinhibition, and interactions with other proteins or lipids [15,16]. As was documented, this interaction occurs in the vascular endothelium in the GTPase cycle [16]. It is worth mentioning that defects in vascular endothelial cells have been often associated with periventricular leukomalacia and with the symptoms observed in the examined patient, encompassing the inflammation and white matter lesions within the developing nervous system [38,55,56,57]. Additionally, the GTPase cycle was previously associated with PVL, neurodevelopmental delay, cerebral palsy and hyperoxia-induced neonatal brain injury [58,59,60]. Specifically, Rho family GTPases operate as molecular switches, cycling between inactive GDP-bound and active GTP-bound forms, regulated by factors like guanine nucleotide-exchange factors and GTPase-activating proteins [61,62,63]. As documented, these processes’ defects may lead to periventricular leukomalacia [6,59,64]. The PEPPI algorithm predicted a reduced interaction between the mutated PLEKHG1 and CDC42, with a score of 1.072, compared to a score of 1.083 for the wild-type PLEKHG1. Indeed, we hypothesize that the observed variant within the PLEKHG1 protein may disrupt its interaction with CDC42 in the GTPase molecular pathway, leading to dysregulation.

Based on these hypotheses, we emphasize that the variant identified can significantly contribute to determining a patient’s phenotype in addition to environmental factors. Despite the potential associations of preterm birth and cotwin fetal abortion with conditions like cerebral palsy and periventricular leukomalacia (PVL), evidence in the literature and the potential involvement of the PLEKHG1 protein pathway with PVL support its role in the patient’s symptoms.

Functional analyses based on in vitro models are required to investigate the specific role of PLEKHG1 in PVL. Nevertheless, the purpose of this work was to lay the foundation for additional studies to individuate the genetic causes related to periventricular leukomalacia and white matter hyperintensities.

The findings and their implications should be discussed in the broadest context possible. Future research directions may also be highlighted.

## 5. Conclusions

In the present study, we present a clinical case of a patient displaying periventricular leukomalacia as an outcome of a perinatal hypoxic-ischemic event. The patient was born prematurely and was the cotwin of a spontaneous abortion in a dichorionic pregnancy. WES Trio analysis identified a heterozygous de novo variant within *PLEKHG1*, a gene previously linked with white matter hyperintensities. The genetic variant spanned into the DH functional domain, which serves as an essential catalytic site. As previously documented, the DH domain is an essential activator of CDC42, a small GTPase associated with PLEKHG1 in GTP regulating different functions in the GTPase cycle. Genetic variants within *CDC42* have been associated with an MIM phenotype code for a wide range of symptoms, including white matter abnormalities. As outlined by previous studies, *PLEKHG1* is ubiquitously expressed, showing high expression patterns in diverse brain regions. We posit that the identified variant may contribute to PVL alongside environmental factors such as preterm birth and cotwin fetal abortion, with implications supported by evidence in the literature and the protein pathway’s association with periventricular leukomalacia (PVL). The aim of this study was to expand the knowledge regarding the genetic basis related to PVL, a debilitating condition impacting newborns. In particular, our purpose was to deepen understanding of the role of the *PLEKHG1* gene, laying the basis for further functional studies.

## Figures and Tables

**Table 1:** Multiple in silico predictions related to the variant c.370A>G, p.Thr124Ala within *PLEKHG1* (NP_001025055.1) gene.

Tool	Significance	Score
MetaRNN	Pathogenic Strong	0.9629
BayesDeladdAF	Pathogenic Moderate	0.3782
BayesDelnoAF	Pathogenic Moderate	0.3055
REVEL	Pathogenic Moderate	0.906
MetaLR	Uncertain	0.7318
MetaSVM	Uncertain	0.7608
CADD	Pathogenic Supporting	27.3
EIGEN	Pathogenic Moderate	0.9774
EIGEN PC	Pathogenic Moderate	0.8997
Mutation assessor	Pathogenic Moderate	3.705
MutPred	Pathogenic Moderate	0.855
FATHMM-MKL	Pathogenic Supporting	0.9912
LIST-S2	Pathogenic Supporting	0.9889
LRT	Pathogenic Supporting	0
MVP	Pathogenic Supporting	0.9612
PolyPhen2	Probably Damaging	0.998
SIFT	Pathogenic Supporting	0.001
DEOGEN2	Benign Supporting	0.3109
BLOSUM	Uncertain	−1
DANN	Damaging	0.9981
FATHMM	Tolerated	−1.1
FATHMM-XF	Damaging	0.8462
M-CAP	Damaging	0.1503
MutationTaster	Disease causing	0.9999
PrimateAI	Tolerated	0.7779
PROVEAN	Damaging	−4.37
SIFT4G	Damaging	0.007

## Data Availability

The data presented in this study are in the main text and in the Supplementary File. Further data are available on request from the corresponding author.

## Supplementary Materials

The following supporting information can be downloaded at: https://www.mdpi.com/article/10.3390/genes15081096/s1. Table S1: ACMG criteria. Figure S1: Heatmaps generated as results of the AlphaFold prediction for determining the best predicted model of the mutated PLEKGH1. Figure S2: Scatterplot related to the pathogenic prediction algorithm score employed for the variant classification. Figure S3: Prediction of the most conserved region by sequence coverage analysis. Figure S4: Predicted Local Distance Difference Test (plDDT) score for PLEKHG1.
