# Supplementary material for: PLEKHG1: New Potential Candidate Gene for Periventricular White Matter Abnormalities
Source: Genes (Basel). 2024 Aug 20;15(8):1096. doi: 10.3390/genes15081096 (PMC11353482; doi:10.3390/genes15081096)
Supplement: Supplementary file 1 [file genes-15-01096-s001.zip › genes-3123348-supplementary.pdf]

Table S1. American College of Medical Genetics (ACMG) criteria adopted for the classification of the identified nucleotide variation within *PLEKHG1* gene as likely pathogenic.

Criteria for classifying variants)	*Category code*	*Description*
Strong	PS2	De novo (both maternity and paternity confirmed) in a patient with the disease and no family history.
Moderate	PM2	Absent from controls (or at extremely low frequency if recessive) in Exome Sequencing Project, 1000 Genomes Project, or Exome Aggregation Consortium.
Supporting	PP3	Multiple lines of computational evidence support a deleterious effect on the gene or gene product
ACMG variant classification		Likely Pathogenic

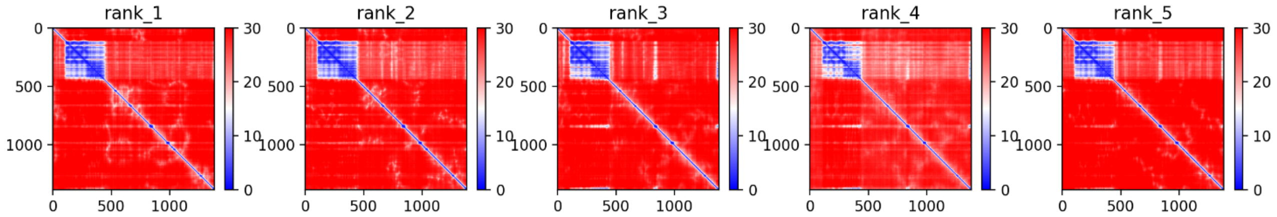

Figure S1. Heatmaps generated as results of the AlphaFold prediction for determining the best predicted model of the mutated PLEKGH1. Specifically, the diagonal line from the bottom-left to the top-right represents the proximity of residues to themselves, which is always close and thus should be present in all maps. Blue regions indicate contacts between residues. Larger and more defined blue regions typically indicate a higher confidence in the structural prediction for those parts of the protein. Red regions indicate areas where there is no contact between residues.

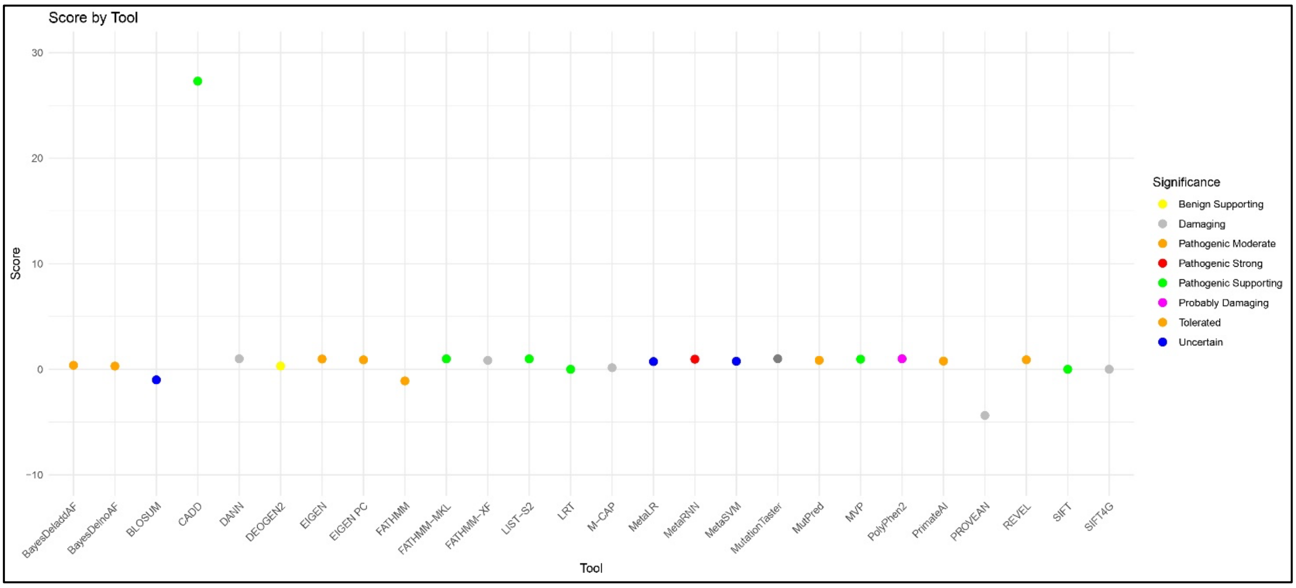

Figure S2. Scatterplot related to the pathogenic prediction algorithms score employed for the variant classification. The different colours indicated in the legend indicate the different prediction based on the score. The plot was realized using R studio version 3.4.3. with the package ggplot2.

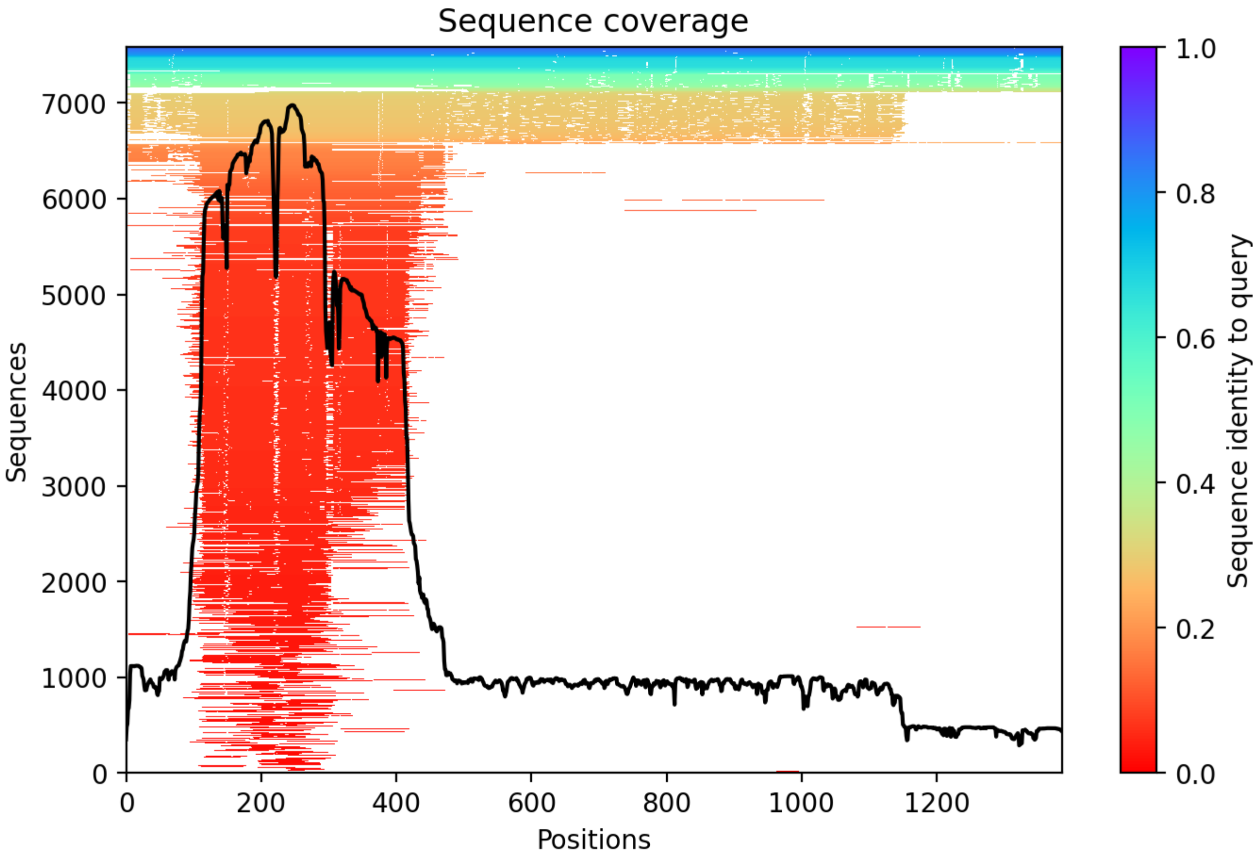

Figure S3. Prediction of the most conserved region by the sequence coverage analysis. The alpha fold analysis compared the whole protein sequence across more than 7000 sequences. The x-axis represents the position of the amino acids in the query sequence. Each number corresponds to a specific residue position or range of positions in the query sequence. The y-axis represents the number of sequences aligned to the query sequence. The black line likely represents the average or cumulative coverage across these positions, indicating the density or number of sequences aligning at each position. As is clearly depicted in the Figure, the specific mutation site at position 124, is located within a conserved region.

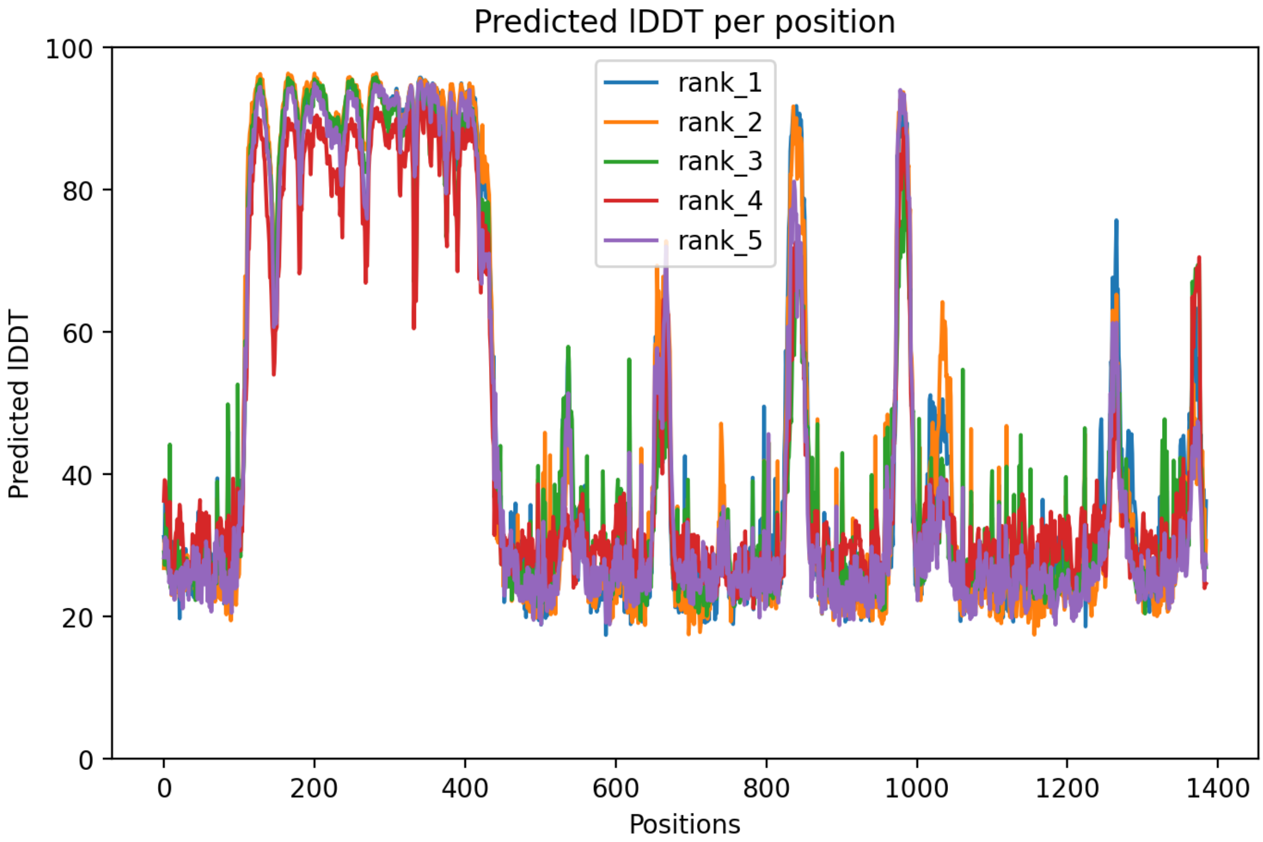

Figure S4. Predicted Local Distance Difference Test (plDDT) score for PLEKHG1. This is a confidence metric used in protein structure prediction, particularly with AlphaFold, employing UCSF ChimeraX. The plDDT score, ranging from 0 to 100, measures the confidence or accuracy of the predicted protein structure at a local, residue-by-residue level, with higher scores indicating greater confidence.
